# An E-Learning Program for Increasing Physical Activity Associated Behaviors Among People with Spinal Cord Injury: Usability Study

**DOI:** 10.2196/14788

**Published:** 2019-08-21

**Authors:** Jereme D Wilroy, Kathleen A Martin Ginis, James H Rimmer, Huacong Wen, Jennifer Howell, Byron Lai

**Affiliations:** 1 Department of Physical Medicine & Rehabilitation University of Alabama at Birmingham Birmingham, AL United States; 2 UAB/Lakeshore Research Collaborative School of Health Professions University of Alabama at Birmingham Birmingham, AL United States; 3 Department of Medicine Division of Physical Medicine & Rehabilitation University of British Columbia Vancouver, BC Canada; 4 School of Health & Exercise Sciences University of British Columbia Kelowna, BC Canada; 5 International Collaboration on Repair Discoveries University of British Columbia Vancouver, BC Canada; 6 Centre for Chronic Disease Prevention and Management University of British Columbia Kelowna, BC Canada; 7 Department of Physical Therapy School of Health Professions University of Alabama at Birmingham Birmingham, AL United States

**Keywords:** physical activity, mhealth, ehealth, people with disabilities, spinal cord injuries

## Abstract

**Background:**

The majority of people with spinal cord injury (SCI) in the United States are not meeting the recommended guidelines for regular physical activity. Behavior change techniques (eg, goal setting and action planning) that are framed within the principles of the social cognitive theory (self-efficacy and self-regulation) have the potential to enhance physical activity behavior.

**Objective:**

The aim of the study was to develop and test the usability of an electronic learning (e-learning) program for improving social cognitive factors related to physical activity behavior among people with SCI.

**Methods:**

The program was created through an iterative process of development and refinement, using a modification of a similar methodology used to develop evidence-informed guidelines in health promotion for people with disabilities (Guidelines, Recommendations, and Adaptations Including Disability; GRAIDs framework). The study included 4 phases: (1) initial product creation, (2) national survey, (3) expert review, and (4) usability testing. Usability testing included both quantitative and qualitative data collection and analyses.

**Results:**

The review of the program by an expert panel (n=5) and the results from a national survey (n=142) led to several refinements. Usability testing demonstrated that the program could be completed in a timely manner (<30 min). Participants reported 5 themes: (1) the program improves social cognitions related to physical activity participation; (2) reflection of physical activity behavior; (3) positive perceptions of the quality of the program; (4) positive perceptions of the program operation and effectiveness; and (5) recommendations for improvement. Each item was incorporated into a revised program version 1.0.

**Conclusions:**

This study incorporated an evidence-based framework for developing a brief 30-min e-learning program for increasing the physical activity behavior among people with SCI. The Exercise Strategies Through Optimized Relevant Interactive E-learning Storytelling (e-STORIES) program could be completed in a timely manner and was reported by participants as valuable and useful for enhancing intent-to-perform physical activity in individuals with SCI. The program has the potential to be applied in a variety of settings, but feasibility testing is required before implementing in a larger trial.

## Introduction

### Background

Regular physical activity participation is a core component of the postrehabilitative care among people with spinal cord injury (SCI). Over 30 years of research has indicated that people with SCI can increase one or more aspects of their physical and mental health through participation in physical activity [1,2]. Physical activity participation can further reduce the risk of chronic disease [3] and other secondary medical conditions [4]. Nevertheless, reports have indicated that the majority of people with SCI do not participate in sufficient amount of leisure-time physical activity (LTPA) to achieve health benefits [5-7].

Individuals with SCI are often overwhelmed by a multitude of barriers [8] that prevent them from participating in LTPA (ie, scheduled activity done in their free time, such as exercise or sport). These barriers can include the lack of transportation, time, and knowledge and the psychological factors such as lack of intrinsic motivation and negative attitudes or beliefs [8]. Negative perceptions can include the belief that the society does not perceive people with SCI as capable of participating in LTPA, that engaging in exercise can cause pain or injury, or that preinjury physical activities are no longer available. These challenges support the need to develop convenient educational programs that can effectively increase the LTPA behavior among individuals with SCI.

Interventions aimed at increasing LTPA in people with neurological disabilities have been found to be more effective when framed within a theory of behavior change [9]. In particular, the social cognitive theory (SCT) is an established and commonly applied theory for promoting physical activity among people with disabilities [2,10]. A strength of SCT over other models of health behavior is that it specifies predictors and principles that can be used for informing, enabling, guiding, and motivating individuals to independently modify (ie, self-regulate) their health behaviors [11]. Specific SCT-based techniques that can be utilized by LTPA promotion programs for persons with SCI include coping with barriers, monitoring behavior, setting goals, and action planning [9,12-14].

One method that may be effective for communicating physical activity information is through story-based communications [15-18]. Perrier et al [16] have suggested that aspects of narrative inquiry may explain LTPA behavior among people with SCI, in addition to the theory-based determinants. Story-based communications capitalize on the narrative tradition, whereby people are recognized as story-telling beings who make sense of their life events and lives by hearing and sharing stories [19,20]. Although a story is characterized by personal communication (ie, what is actually being told by a person), narrative differs insofar as it is characterized by the properties, such as themes or structures, that comprise the stories [20].

Preliminary data suggest that storytelling might be an effective strategy for conveying physical activity information to people with SCI [18,21]. Furthermore, qualitative inquiry suggests that storytelling may be more effective when it is tailored to the individual’s *narrative style* [16]. Within the context of SCI and physical activity, narrative style refers to how a person with SCI perceives LTPA impacting their personal life story. Using narratives first developed by Arthur Frank [20], 3 key narrative styles have been identified and adapted to the narrative inquiry with regard to SCI and LTPA [16,17]: (1) chaos narrative (lack of future and focus on preinjury LTPA), (2) restitution or cure narrative (LTPA as a means to restore former self), and (3) quest narrative (LTPA as an opportunity for social involvement, health benefits, and enjoyment postinjury). The goal of incorporating these narrative styles into a storied communication about LTPA is to connect with individuals with SCI who identify with a particular narrative, and to thereby promote examination of their own thoughts concerning their LTPA behavior [16]. However, these narratives have not yet been embedded and tested within a story-based, electronic learning (e-learning) program developed for people with SCI.

E-learning technologies, combined with health behavior theory and narrative style, have the potential to provide educational opportunities to teach people how to overcome barriers to LTPA. E-learning authoring software (eg, Articulate 360) can be used to deliver easily accessible, interactive, and personalized educational programs. With the guidance of a narrative, individuals can be the narrators of their own e-learning storyline that adapts to their responses from programmed questions and queries. E-learning narratives can be combined with behavior change activities and engaging, visually attractive content (eg, text, pictures, audio, animation, and video) to modify SCT determinants, enhancing the likelihood of an individual engaging in LTPA. Although there are long-term behavioral coaching programs that can increase engagement in LTPA [2], there are limited educational programs that are short in duration and can be implemented in time-sensitive settings (eg, clinic waiting rooms) or disseminated on a national level.

### Objective

The aim of this study was to develop and test the usability of a brief e-learning program that embedded SCI narratives in a story-based approach, with the goal of improving SCT constructs that are related to LTPA. Program development included immersive involvement by people with SCI and input from an expert panel of researchers. This paper describes the development and usability of the program e-STORIES (Exercise Strategies Through Optimized Relevant Interactive E-learning Storytelling).

## Methods

### Overview

**Figure 1:**
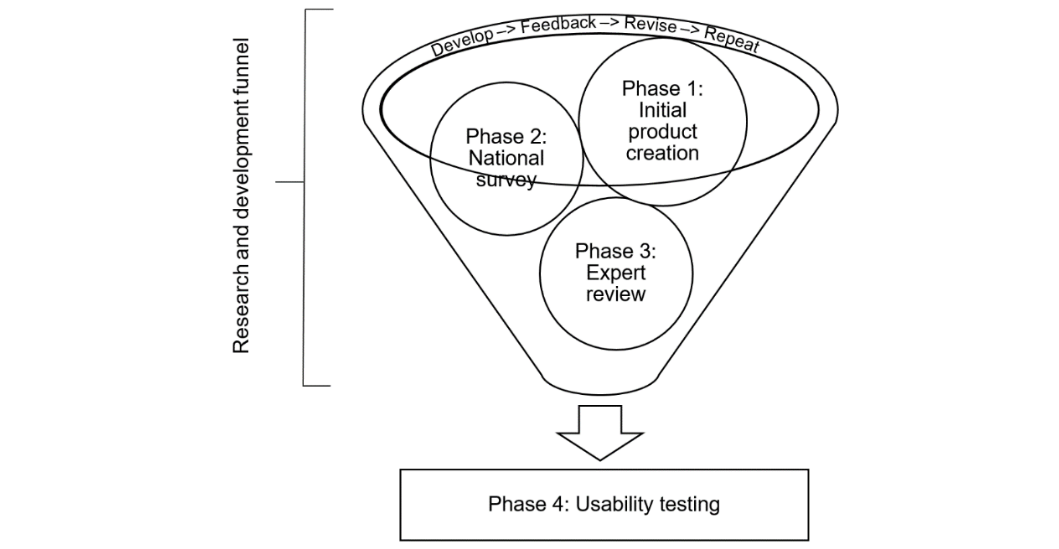
Research and development process for spinal cord injury Exercise Strategies Through Optimized Relevant Interactive E-learning Storytelling program.

The e-STORIES development process was adapted from the Guidelines, Recommendations, and Adaptations Including Disability (GRAIDs) framework [22]. GRAIDs was chosen as a guiding framework as it is a systematic series of steps for developing or modifying health promotion programs or guidelines for people with disabilities and has been used to develop at least 11 programs thus far [22]. Following this framework, the e-STORIES program was developed through 4 phases: (1) a review of the literature to inform initial development, (2) a national survey of people with SCI, (3) an expert panel that reviewed and vetted the content material, and (4) usability tests among a sample of people with SCI. These phases went through an iterative research and development process of *develop, feedback, revise, and repeat* (see Figure 1).

### Phase 1: Initial Product Creation

Initial development of the computer-based program involved 4 elements: literature review, storyboard creation, theory and intervention alignment, and creation of the infrastructure for the program.

#### Literature Review

A systematic review of peer-reviewed publications was conducted using the search terms: “spinal cord injury” AND “physical activity” AND “behavior change” AND “self-management.” The study inclusion criteria included studies that were (1) peer-reviewed, (2) conducted within the past 2 years (2016-2018), (3) classified as a review paper, and (4) indexed in PubMed, Cumulative Index to Nursing and Allied Health Literature, or Scopus. The search for review papers returned a total of 10 articles and no duplicates. After screening these articles for eligibility criteria there was only 1 article remaining. Other articles were removed owing to not being a systematic review or not targeting SCI population.

The single systematic review of physical activity self-management interventions for people with SCI was used to identify behavioral LTPA interventions and strategies to aid in content development for the e-STORIES program [23]. This report identified 31 unique studies published from 1980 to 2015. On the basis of the review of these studies and the consideration of the SCT constructs, the project team incorporated into the e-STORIES program, the behavior change techniques [12] of goal setting, problem solving, and action planning. After the completion of the storyboard and the basic infrastructure for the e-learning product, the behavior change techniques and narratives were incorporated to generate program version 0.1.

#### Storyboard Creation

The first stage of development was to create a storyboard that outlined the flow of the program, content suggestions, and scripts, as well as naming the project and designing a logo. The storyboard displayed the flow of the e-learning intervention and whether the information would be delivered by audio, text, pictures, animation, video, or some combinations of these media. The storyboard also helped with the construction of the pathways for tailored survey responses (ie, the flow of the program would adapt, based on a participant’s responses, to items related with confidence and self-regulatory strategies such as goal setting or planning).

#### Theory and Intervention Alignment

SCT is based on the interplay between the behavioral factors, personal factors, and environmental factors [24]. The intervention was framed within SCT such that the intervention content was designed to target, and improve, the SCT constructs of self-efficacy, self-regulation, and outcome expectations. This was achieved by providing the participants with activities that taught them how to set Specific Measurable, Achievable, Relevant, Time goals, action plan, problem-solving barriers (targets self-regulation constructs of SCT), identify true and false outcomes of LTPA (targets outcome expectations), and view video content of others with SCI performing LTPA (targets vicarious experience).

#### Program Infrastructure

Storyline 360, developed by Articulate Global Inc, is an e-learning authoring tool that was used to create the e-STORIES program. This software allowed the program to be interactive and instructional through multimedia that included digital movies, pictures, sound files, animation, graphics, and other electronic files. After a story board was created, the basic outline of the program was developed in Articulate’s Storyline 360. The outline included (1) program introduction, (2) assessment of theory constructs, (3) LTPA behavior change activities, and (4) printed copy of the participant’s results.

### Phase 2: National Survey

The second phase of the study included a survey of individuals with SCI that was delivered on the Web using Qualtrics. The survey aimed to collect descriptive information on the physical activity readiness and identification with SCI narratives related to LTPA, to inform and revise the program. Participants were asked to indicate which of the following best described their current level of physical activity: (a) Yes, I have been doing LTPA regularly for more than 6 months; (b) Yes, I have been doing LTPA regularly for less than 6 months; (c) No, but I intend to start doing LTPA regularly in the next 30 days; (d) No, but I intend to start doing LTPA regularly in the next 6 months; or (e) No, and I do NOT intend to start doing LTPA regularly in the next 6 months [6].

A description of each of the three SCI LTPA narrative styles was developed from the literature [16,20]: (1) LTPA is not important to me and it reminds me of how I am not able to do things like before my SCI (chaos); (2) LTPA helps to improve function and maintain my physical health for a potential cure (cure); or (3) LTPA helps me through life and helps me connect with others similar to me (quest). The survey ended with 2 open-ended questions that asked participants for desired features and messages: (1) What do you think should be included in an educational program that aims to increase physical activity among people with SCI? and (2) What would you tell another person with an SCI if you were trying to help them become more physically active?

#### Participants

People with SCI were recruited through one of 12 SCI-related support groups or organization pages on a social media platform. The eligibility criteria included participants who (1) were aged 18 years or above, (2) had a diagnosis of SCI, (3) had the ability to use hands and arms to exercise, and (4) had the ability to converse in English and complete the survey forms.

#### Analyses

Using a measure of physical activity readiness, participants were categorized as physical activity nonintenders, intenders, or actors [25], to assess the differences of physical activity readiness among the SCI narratives [6,25]. This hypothesis was based on previous literature [17,21], and if true, one programming strategy could involve grouping the physical activity readiness with SCI narratives. Fisher exact test was used to assess the differences in categorical variables (sex, injury level, and physical activity readiness) by narrative groups. Owing to the nonnormal distribution of the numerical variables (age and years since injury), Kruskal-Wallis test was used to examine the differences in age and years since injury by narrative groups. SAS version 9.4 was used for statistical analysis and statistical significance was set a priori at *P*<.05.

### Phase 3: Expert Panel Review

An expert panel reviewed each component of the e-learning program for quality and accuracy using Articulate’s 360 Review, which allowed them to review the program online and add comments. The panel included an exercise psychologist, an exercise physiologist, a rehabilitation scientist, an information specialist, and a clinician who had several decades of experience in exercise research in SCI. After completing the review, each expert provided a summary of suggested revisions. This feedback led to several revisions, including textual, audio, and video changes and adding features to better guide participants. This phase included program *versions 0.2 and 0.3*.

### Phase 4: Usability Testing

#### Study Design

The usability testing incorporated a nested mixed-methods design (quantitative+qualitative), where the qualitative component was embedded within a primarily quantitative study design. In other words, the usability study included a qualitative interview within a design that emphasized the core fundamentals of usability testing. Quantitative and qualitative aspects were treated with equal value. On the basis of the best practice recommendations for usability testing [26], a mixed-methods design was chosen to provide a comprehensive evaluation of usability in 4 core areas: effectiveness, efficiency, satisfaction, and usefulness.

Belief systems in the mixed-methods studies are critical as they influence all aspects of a study, including the design, analyses, and even the presentation of the results [27]. Separate belief systems (ie, philosophical assumptions) guided each method of this study until data were interpreted within the discussion, which allowed the research team to adhere fully to the quality and rigor demanded by both methods. This approach was aligned with the paradigm of dialectical pluralism [28]. Dialectical pluralism is a metaparadigm that calls for the use of separate belief systems for both the quantitative and qualitative aspects of a mixed-methods study, particularly when the study aims to give equal emphasis to both methods. The quantitative study components aligned with the positivist perspective: reality and knowledge are singular and, thus, every event within the world can be scientifically quantified. For example, statistically significant results inform us of an event that has occurred. The qualitative study component was underpinned by an interpretivist philosophical approach: reality is multiple and subjective (ie, ontological relativism) and knowledge is socially constructed (epistemological subjectivism) [29]. For example, the research team acknowledged that the qualitative results would be created through the interaction of the interviewer and interviewee. Therefore, the interviewer did not ask questions as a blind observer. Instead, the interviewer understood that the participants’ responses could be probed with additional follow-up questions to create a richer dataset (based upon the insight of the interviewer).

#### Recruitment

This study aimed to enroll 12 inactive people with SCI to satisfy the best practice recommendations for usability testing [26]. People were eligible if they were (1) aged 18 years or above, (2) diagnosed with an SCI, (3) achieving less than 60 min of moderate or vigorous intensity exercise per week, (4) able to speak and understand English, and (5) able to operate a computer. This project was approved by the Institutional Review Board of the university. Before the enrollment, written consent was obtained from each participant.

#### Procedure

Participants were briefed about the study and provided the written consent forms. After obtaining the consent, participants’ demographics and clinical characteristics were recorded. Participants were then instructed to complete 16 usability tasks which involved navigating through and completing the entirety of the program and responding to the interactive questions. A research assistant took written notes while observing the participants perform the usability tasks and recorded the time taken by the participants to complete the entire e-learning program (all tasks). This was followed by a one-on-one interview conducted in a private and comfortable setting within the research laboratory. Examples of the interview questions included: “Describe to me some positive experiences of the program,” “Describe to me some negative experiences or issues you experienced with the program,” and “Tell me your overall thoughts about the program.” The interview was recorded by an audio device, which was later transcribed for qualitative analysis.

#### Measures

Usability was defined in terms of effectiveness (ie, the ease at which individuals can use the product), efficiency (ie, the speed at which an individual can accurately complete a task), usefulness (ie, the extent a product can enable the users to achieve their goals and the willingness to use the product), and satisfaction (ie, the users’ perceptions and opinions of the product). Usefulness and satisfaction were explored through qualitative means, whereas effectiveness and efficiency were examined through quantitative metrics.

##### Effectiveness

Effectiveness was measured as the number of slides on which participants experienced an issue or problem, divided by the total slides that were completed without issues, which resulted in a single percentage value. The research team set an a priori benchmark of acceptable effectiveness at 90% [26].

##### Relative Efficiency

Relative efficiency was measured by the time required to complete the entire module, which included the 16 tasks. The research team established a benchmark of acceptable efficiency at 30 min, which was 8 min longer than it took the lead researcher to complete the module. The program was developed to be brief so that it could be used during clinic visits (eg, waiting periods) or at a baseline visit for someone starting an exercise intervention.

##### Usefulness and Satisfaction

Researchers assessed the usefulness and satisfaction through participants’ qualitative feedback from the face-to-face interviews. Each interview included open-ended questions that sought to gain insight into the participants’ overall perceptions of the module, their likes and dislikes regarding module features and content, whether they would use the module if it was provided to them in the clinic, and whether they perceived the information as valuable. One member of the research team conducted the interviews. The interviewer had 2 years of experience with qualitative research that involved usability testing of exercise technology.

#### Analysis

Participant characteristics and quantitative usability data were descriptively reported. Qualitative data were analyzed using latent deductive thematic analysis (ie, a thematic analysis process was used in which data were analyzed with the intent of developing a surface level description of the 4 core areas of usability) [30]. Thematic analysis was conducted by 2 researchers who were guided by the 6 steps proposed by Braun and Clarke [31]. First, the analysts independently read through the transcribed interviews and listened to the verbal recordings when the meaning of the written text was ambiguous (familiarization). Second, the analysts generated initial codes from segments of the transcribed interviews. Third, the analysts refined these codes into fewer subthemes and repeated this process for each transcription. Fourth, the analysts then met to review their subthemes, which they then integrated and refined into a single set of themes. Fifth, the analysts then defined and named each of these themes. Sixth, the resultant themes were reported. To enhance the quality of these data, the analysts functioned together as critical friends [32,33] and conducted the analysis within the aforementioned interpretivist philosophical approach. Both analysts had training and experience in the mixed-methods research and exercise training for people with SCI. One analyst was the principal investigator who was a physically active person living with SCI and believed in the value of LTPA. The other analyst had a background in adapted physical activity.

## Results

A total of 154 people with SCI contributed to the development of the program. The national survey was completed by 142 participants, and 12 people enrolled in and completed the usability testing.

### National Survey

Participant characteristics for the national survey are shown in Table 1. Data for age and years since injury were comparable with national estimates, but there was a slightly higher representation of females than that which has been reported nationally [34]. There were 61.3% (87/142) participants that identified with the cure narrative, 30.2% with quest narrative, and 8.5% with the chaos narrative. The finding that the majority of the sample identified with a cure narrative is in line with previous research [17]. Fisher exact test indicated no significant differences (*P*=.18) in physical activity readiness across the narrative groups. The open-ended questions were compiled into a list and redundancies removed to provide a list of suggestions to include in the program. Overall, the analysis of the national survey data resulted in the addition of video content of active people with SCI who shared their stories, videos of adapted exercises and sports, and emphasis of health benefits associated with LTPA.

**Table 1:** Results of national survey (N=142).

Characteristics		Values
Age (years), mean (SD)		42 (14)
Years since injury (years), mean (SD)		12 (12)
**Gender^a^, n**			
	Male	71
	Female	55
**Level of injury, n**			
	Paraplegia	91
	Tetraplegia	51
**Narratives, n**			
	Cure	87
	Quest	43
	Chaos	12
**Physical Activity readiness, n**			
	Actor	115
	Intender	22
	Nonintender	5

^a^Data from 16 participants are unreported.

### Expert Panel

The following summarizes the revisions completed after the expert review: a statement about pain and exercising as a person with SCI was changed; some videos were re-recorded to improve quality; volume was increased on several audio clips; minor text edits were made; option was added in narratives for participants to use their own words or phrase; and examples of goals and next steps were provided.

### Usability Study

Usability testing of program *version 0.4* led to several minor revisions that were incorporated within the final program *version 1.0*. In total, participants experienced 29 usability issues. The issues were related to (1) operating action prompts during a task (24/29, 83% of issues), (2) operation errors (a broken navigation button that linked to the previous page; 2/29, 7% of issues), (3) verbal clarity (2/29, 7% of issues), and (4) video resolution (1/29, 3% of issues). Table 2 provides demographics for participants in the usability study.

**Table 2:** Participant demographics of usability study (N=12).

Characteristics		Values
Age (years), mean (SD)		53 (11)
Years since injury (years), mean (SD)		23 (13)
**Gender, n**			
	Male	8
	Female	4
**Level of injury, n**			
	Paraplegia	12
	Tetraplegia	0

#### Effectiveness

The mean effectiveness score was 86% (SD 10%; 95% CI 80%-91%). This value was slightly lower than our a priori benchmark of 90%, indicating that minor changes were required to reduce operation issues.

#### Efficiency

The mean time to complete the program was 28 min 20 seconds (SD 6 min 45 seconds; 95% CI 25 mins 43 seconds to 33 min 3 seconds), which was lower than our a priori benchmark of 30 min.

#### Usefulness and Satisfaction

The analysts identified 5 themes regarding the usability of the program: (1) the program promoted reflection/introspection of physical activity behavior; (2) the program improved social cognitions related to physical activity participation; (3) the participants had positive perceptions of the quality of the program; (4) the participants had positive perceptions of program operation and effectiveness; and (5) participant recommendations for improvement. These themes and their subthemes and supporting quotes are shown in Table 3.

Participants reported that the program promoted valuable thoughts of self-reflection regarding physical activity behavior. The program provided relevant and encouraging information to establish reasonable outcome expectations of LTPA and to progress from a focus on the injury to resuming normal daily activities as reported by participant 1: “It definitely gets you from being focused on the actual injury to getting back into life, getting that groove back into life.”

Participants reported that the program improves social cognitions related to physical activity participation such as self-regulation. The program increased the participants’ confidence to plan and schedule PA through behavior change techniques that included goal setting, demonstration of the behavior, and how to plan physical activity around barriers that prevent participation. Participants further noted that the content provided meaningful and relevant information, with an uplifting and motivating tone:

It gets you to thinking. It gets you out of the doom and gloom process, and into the hopefulness, the looking forward to...And that's why I see it as being a major help in that area.

Participant 2

Participants reported high levels of satisfaction with the program design, including the animations and general flow of the slides. Participants further described the program as self-explanatory, intuitive, easy to operate, and engaging. However, the participants also had recommendations for improving the usability of the program. Participants noted that minor changes in the form of visual cues or instructional prompts and a back button could assist navigation and clarity in areas of the program that caused confusion.

**Table 3:** Qualitative results of usability study.

Themes and subthemes		Supporting quotes
**The program promoted reflection/introspection of physical activity behavior**		
	The program provided relevant and encouraging information that fostered thoughts of hope	"It gets you to thinking. It gets you out of the doom and gloom process, and into the hopefulness, the looking forward to...And that's why I see it as being a major help in that area."[Participant 002]
	The program provided valuable suggestions to progress from a focus on the injury to resuming normal daily activities	“It definitely gets you from being focused on the actual injury to getting back into life, getting that groove back into life.” [Participant 001]
**The program improves social cognitions related to physical activity participation**		
	The program enhanced participants’ intentions to plan and schedule physical activity.	“It was very valuable because, like I said, I'm going to apply this to my everyday life from now on, and I'm going to start setting more planned goals and put them into motion.” [Participant 009]
	Goal setting and vicarious experiences were memorable techniques that were learned from the program	“I think the positive is kinda relaying how you would go about obtaining a goal, like the time aspect of it. Set it 10 minutes per day, 30 days. And, the stories about the spinal cord injuries. Just seeing somebody else overcome the journey you've already been on.” [Participant 005]
**Positive perceptions of the quality of the program**		
	The content provided information that was meaningful and relevant to the individual	“It's really a new day about spinal cord injury, and it show a lot about peer support when you're a spinal cord injury. It shows a lot about activities, it shows about people who've had trauma in their life and who have overcome the trauma, and they're living a very high functional life.” [Participant 002]
	Participants were satisfied with the program design	“That was awesome and I liked the way he told his story about what happened to him. Some of the drawings and the way they did, I thought that was pretty good. The animation, the way they did that. I thought that was good.” [Participant 013]
	Perceived as a valuable tool to enhance physical activity behavior for people with acute spinal cord injury	“Just that, I think, it would be beneficial for anyone with a spinal cord injury to participate in it or see it, especially somebody newly injured.” [Participant 008]
	The content had a tone that was uplifting and motivating	“Very uplifting. I loved the manner in which the information was presented. It was very positive. It was motivating.” [Participant 008]
**Positive perceptions of program operation and effectiveness**		
	The program was self-explanatory and intuitive	“It kind of explained what it wanted you to do. You just clicked on it and it told you.” [Participant 011]
	The program was easy to operate	“I guess with the mouse, with the clicking...The mouse helped me navigate through it easier and stuff, instead of using the pad.” [Participant 001]
	The program was engaging	“It's quite interactive, and well thought out I will say. It's easy to navigate and keeps you into it. So, it's very much needed for a lot of people to get back into feeling the need for this type of program.” [Participant 009]
**Recommendations for improvement**		
	Improve visual cues/prompts to assist navigation	“The navigation was not bad, but it’s just...do I click on the box or do I move it? So that was a little confusing.” [Participant 001]
	Enhance the definition of the different types of exercise intensities and the action prompts	“The only negative I had was the calculation of the mild to moderate when it changed it from. I entered 30 and it went to 90. It's just a little confusing...Yeah. A different color on the words mild, moderate, heavy. Maybe put them in bold. A different font. Just something to make it stand out so that it's emphasized...Potentially lead with: “You're going to be asked about three different levels of exercise,“ so that you know ahead of time that you'll be asked the same thing three times just differentiating mild, moderate, and heavy.” [Participant 008]
	Provide the ability to go back to a previous screen	“Well not being able to go back to the previous screen if I messed up. I even tried to click next one time and it didn't work either. So that was a negative.” [Participant 006]

### Description of Exercise Strategies Through Optimized Relevant Interactive E-learning Storytelling Version 1.0

**Figure 2:**
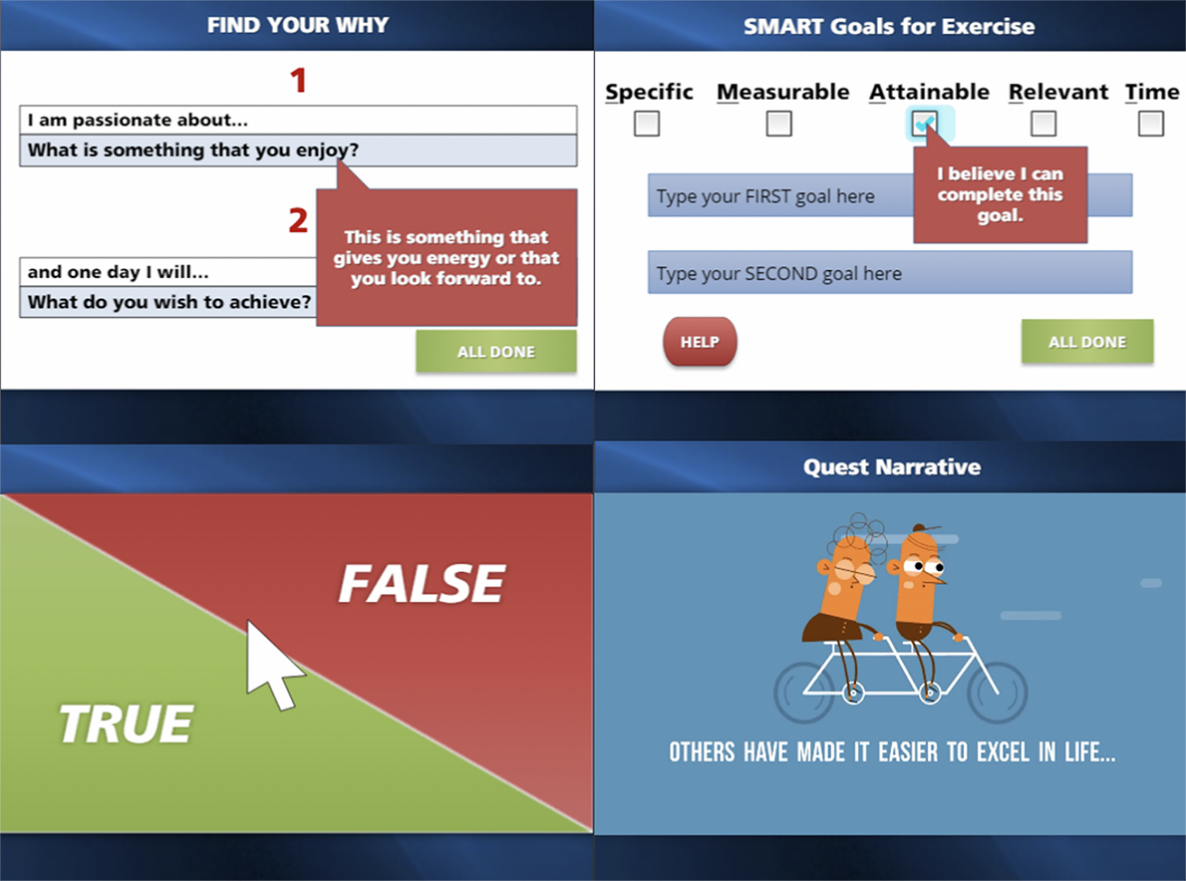
Screenshots of spinal cord Exercise Strategies Through Optimized Relevant Interactive E-learning Storytelling injury program.

The final program version included a total of *41* slides (examples shown in Figure 2). The program further contained *16 interactive tasks*, *12 videos*, and *a summary print-out* (see video of full program in Multimedia Appendix 1). The program begins with an introduction, which includes animated videos and a description of acquiring an SCI, discussion of why exercise and planning is important, introduction of program coach, and information about how to maximize the program. Next section of the program includes some questions concerning LTPA behavior, LTPA self-efficacy, and self-regulatory efficacy. Once completed, the program guides the participants through several activities, including selecting a narrative, debunking myths about exercise and SCI, identifying an intrinsic motivation for LTPA, setting a goal for LTPA with a plan for the next step, and matching common barriers to exercise with solutions. The participant then views a video that includes people with SCI sharing their stories and exercising at a recreational facility. Finally, a print-out summarized each participant’s learning module, which included their physical activity goals with a next step, primary motivation for exercise, LTPA narrative, and reported moderate-to-vigorous LTPA minutes.

## Discussion

### Principal Findings

This project incorporated an evidence-based framework for developing and testing a storied e-learning program to increase physical activity among people with SCI. Usability testing demonstrated that people could independently complete the program in a timely manner and that the program was perceived as valuable and useful for improving confidence to engage in physical activity and associated self-regulating behaviors among people with SCI. Usability testing identified minor operation issues that were rectified in the final version of the e-STORIES program.

A strength of this project was the high value placed on stakeholder feedback. Development of the program included input from over 150 participants with SCI. Such feedback informed critical development decisions, including the decision to not tailor the presentation of the SCI narratives based on the participant’s physical activity readiness and to halt further usability testing. Given that usability testing can be defined in several ways, the determination of an endpoint for usability testing is often a difficult task [35]. In this study, the research team found that a synthesis of both the quantitative and qualitative findings was useful in deciding to halt usability testing. Although several usability issues were identified from the quantitative data (particularly the engagement tasks), participants reported favorable perceptions of the program. Furthermore, participants reported that the issues they experienced could be remedied by minor visual enhancements to facilitate navigation, versus changes in program content or information.

Statistical analysis of the results from the national survey (phase 2) did not support tailoring the presentation of SCI narratives based on the participant’s physical activity readiness. Findings demonstrated no significant differences among the SCI narratives and the physical activity readiness. However, only 8.3% (12/142) of the people identified with the chaos narrative, which likely affected the analyses. This finding is consistent with previous research which reported that less people with SCI identified with the chaos narrative when compared with the cure and quest narratives in LTPA studies [16,17]. Nevertheless, the qualitative results indicated that people with SCI found the content relatable and viewing narratives seemed to encourage introspection, or self-examination of thoughts, of LTPA.

Although usability results suggested that the e-STORIES program could potentially improve self-efficacy and self-regulatory skills to perform physical activity, further research is required to explore whether the program can be successfully implemented in pragmatic, real-world settings. One such setting is within the waiting area of a rehabilitation hospital for SCI patients, where the patient could discuss the e-STORIES results with their physician, as people with SCI perceive doctors as a trusted and credible source of PA information [36]. In addition, several of the participants who completed the usability study indicated that the program was ideal for individuals with newly acquired SCI, so another potential setting could be inpatient clinics for people with SCI. Given that all but one participant of the usability study had SCI for 11 or more years since the time of injury, it would be beneficial to test the program among people with recently acquired SCI. A strength of the e-learning program is its potential to reach a broader sample of people with SCI, as e-STORIES could be disseminated globally through internet-distribution channels such as the National Center on Health, Physical Activity and Disability. Finally, the e-learning program could be utilized as a supplementary behavior change tool in an exercise training regime.

### Limitations

This project had a few limitations. Although the sample size for usability testing met 1 minimum recommendation, the sample limited our understanding of usability within various subgroups of people with SCI. Only people with paraplegia enrolled in the study, and these individuals were, on average, middle-aged. This limitation supports the need to test the program with larger heterogeneous samples of people with SCI, particularly individuals who are in the acute or subacute stage postinjury, a period when the program could be highly valuable as noted by several study participants. Another limitation was the low number of individuals in the chaos narrative, reducing the power to detect differences in readiness among the narratives. Finally, although only 3 narratives were presented in the program (cure, quest, and chaos), it is important to note that there have been several other narratives concerning LTPA among people with SCI in the literature [37], such as *exercise is medicine* and *exercise is progressive redemption*, which were not presented in this study.

### Conclusions

e-STORIES is a brief, online program developed through an iterative process that was informed by the life experiences of more than 150 people with SCI, including the creator of the program (JW). The usability study demonstrated that people with SCI perceived the e-STORIES program as valuable, useful, effective, and of high quality. However, feasibility testing is required before the implementation of the program in a larger trial.

## Appendix

Multimedia Appendix 1 Video of Exercise Strategies Through Optimized Relevant Interactive E-learning Storytelling program.

## Abbreviations

e-learning: electronic learning
e-STORIES: Exercise Strategies Through Optimized Relevant Interactive E-learning Storytelling
GRAIDs: Guidelines, Recommendations, Adaptations Including Disability
LTPA: leisure-time physical activity
SCI: spinal cord injury
SCT: social cognitive theory
